# Effect of Different Breathing Aids on Ventilation Distribution in Adults with Cystic Fibrosis

**DOI:** 10.1371/journal.pone.0106591

**Published:** 2014-09-15

**Authors:** Markus Wettstein, Lorenz Radlinger, Thomas Riedel

**Affiliations:** 1 Physiotherapy Institute, Inselspital, Bern University Hospital and University of Bern, Bern, Switzerland; 2 Bern University of Applied Sciences, Health, Bern, Switzerland; 3 Paediatric and Neonatal Intensive Care Medicine, Department of Paediatrics, Inselspital, Bern University Children’s Hospital and University of Bern, Bern, Switzerland; University of Tübingen, Germany

## Abstract

**Background and objectives:**

We investigated the effect of different breathing aids on ventilation distribution in healthy adults and subjects with cystic fibrosis (CF).

**Methods:**

In 11 healthy adults and 9 adults with CF electrical impedance tomography measurements were performed during spontaneous breathing, continuous positive airway pressure (CPAP) and positive expiratory pressure (PEP) therapy randomly applied in upright and lateral position. Spatial and temporal ventilation distribution was assessed.

**Results:**

The proportion of ventilation directed to the dependent lung significantly increased in lateral position compared to upright in healthy and CF. This effect was enhanced with CPAP but neutralised with PEP, whereas the effect of PEP was larger in the healthy group. Temporal ventilation distribution showed exactly the opposite with homogenisation during CPAP and increased inhomogeneity with PEP.

**Conclusions:**

PEP shows distinct differences to CPAP with respect to its impact on ventilation distribution in healthy adults and CF subjects EIT might be used to individualise respiratory physiotherapy.

## Introduction

Respiratory Physiotherapy (RPT) is often used for the promotion of lung expansion, minimisation of atelectasis and the airway clearing. RPT contains breathing manoeuvres, positioning, mobilisation/ambulation, exercise therapy and different airway clearance techniques (ACTs) using different devices.

Improved mucus clearance is considered essential in optimising respiratory performance (airway obstruction, atelectasis, ventilation inhomogeneity, increased work of breathing) and might reduce the progression of lung disease in Cystic Fibrosis (CF). [1] Some ACTs have been shown to lead to significantly greater sputum expectoration compared to breathing manoeuvres alone [2].

Probably the most frequently used airway clearance devices are the positive expiratory pressure (PEP) devices. [1] They provide a constant backpressure to the airways during expiration. It has been hypothesised that PEP increases gas pressure behind the mucus via collateral ventilation and maintains airway patency by stabilising the airways during expiration, thereby improving mucus clearance. [3], [4] A recent study showed superiority of PEP over high frequency chest wall oscillation as the primary form of airway clearance in CF patients. [5] Ventilation distribution is mainly influenced by factors like gravity (posture), ventilation patterns (tidal volume, flow rate) or breathing manoeuvres (type of respiratory aid) and the quality (age, body weight, genetics) and/or pathology of the lung. [6]–[11] Hence, to increase their efficacy, ACT’s are often combined with positioning and breathing aids such as continuous positive airway pressure (CPAP) or positive expiratory pressure (PEP), in order to benefit from postural drainage effects or changes in ventilation distribution [12], [13].

In healthy lungs, most of the tidal volume is directed to the dependent parts of the lung. [11], [14] In certain lung diseases and in elderly, children or obese patients these findings may be different, due to early airway closure and lung collapse in the dependent lung during expiration. [15]–[17] These gravitational effects have been shown to be enhanced with CPAP. [14] PEP devices may homogenise the ventilation distribution and may counteract the early airway closure [3].

To achieve the optimal combination of position and device for each individual patient, it is essential to be aware of the effects of different breathing aids on ventilation distribution in different body positions.

Conventional techniques to assess ventilation distribution such as multiple breath washout (MBW), radio nuclear lung scans or ventilation MRI are either nearly impossible to perform during RPT or give only an overall measure of ventilation distribution without being able to show regional differences. Electrical impedance tomography (EIT) is a imaging technique for bedside monitoring of ventilation distribution. [18] EIT accurately measures tidal volumes and changes in end-expiratory level and describes regional ventilation distribution by measurement of local impedance change and has the advantage of a high temporal resolution [14], [19]–[22].

With the present work, we aimed to compare the effect of different breathing aids on gravity-dependent ventilation distribution in subjects with CF and healthy adults. We hypothesised that the different devices have variable impact on gravity-dependent ventilation distribution in both groups.

## Materials and Methods

### Ethics Statement

This study was designed as a randomised cross-over study and approved by the ethics committee of the region of Bern, Switzerland. Written informed consent from all subjects was obtained at enrolment.

### Subjects

We investigated 11 healthy physiotherapists (5 female), median (range) age 33 (23–45) years and 9 subjects with advanced CF lung disease (2 female), age 31 (19–48) years. All of them were familiar with the use of breathing aids and RPT. Subjects with CF were recruited from outpatient chest clinics at Bern University Hospital, Switzerland. CF subjects showed a forced expiratory volume in the first second (FEV_1_) of median (range) 41 (16–63)% predicted, whereas forced vital capacity (FVC) was 48 (20–70)% predicted. FEV_1_ and FVC in healthy subjects was 108 (95–118)% predicted and 111 (98–121)% predicted, respectively. In healthy as well as in CF there were no smokers.

### Measurements

For each subject, three repetitive recordings of EIT were performed during spontaneous breathing, CPAP (VPAPIII, Resmed AG, Basel, Switzerland) and PEP (PARI-PEP, PARI GmbH, Starnberg, Germany) in upright and right lateral position with the respective commonly used breathing pattern. CPAP pressure were set to 15 mbar in healthy (similar to PEP pressure) and to 10 mbar for CF (according to the hospital guidelines). CPAP was applied by full face mask, PEP was applied by using a mouthpiece and expiratory time was limited to 7 seconds. Body position and the different devices within each body position were applied in a random order. Randomisation was achieved by using sealed envelopes. In between the measurements was a break of at least one minute to check electrodes. Measurements with the new device/position were started after a stabilisation phase of one minute. Based on our own experience this time has shown to lead to stable conditions.

### Data acquisition and processing

A Goettingen GoeMF II EIT tomograph (CareFusion, Houten, The Netherlands) was used with a frame rate of 13 Hz and a recording time of 45 seconds in combination with self-adhesive ECG electrodes (Blue Sensor T, Synmedic, Switzerland). EIT scans were generated from the collected potential differences and the known excitation currents with weighted back-projection in a 32×32 pixel matrix using the software provided with the EIT device. The EIT signal was low-pass filtered below the cardiac frequency and a cut-off mask of 20% of the maximum standard deviation of the mean impedance change (912 pixels) was used. [19], [23] The proportion of ventilation distributed into the right and left parts of the lung (spatial distribution), relative change in end-expiratory level (EEL), relative change in tidal volume and local filling characteristics (temporal distribution) were calculated adapted to previously-published methods in Matlab R2013a (The MathWorks Inc., Nattick, MA, USA). [14] For the calculation of the temporal distribution the time course of the impedance signal of the region of interest was plotted against the global impedance signal breath by breath. The resulting curve was fitted to the equation I(g) = a * g^FI^+c, where the filling index (FI) describes the shape of the curve. The region of interest is filling faster than the rest of the lung if FI<1 and slower if FI>1 [24].

### Statistics

For spatial distribution EIT results are expressed as the percentage of ventilation directed to the right parts of the lung. For filling characteristics FI-values of the right parts of the lung are reported. Changes in EEL from spontaneous breathing within the same body position were normalised for the impedance change during spontaneous tidal breathing in upright position. Changes in EIT tidal volumes are expressed as percentage change from average EIT tidal volume during spontaneous breathing in the corresponding body position. All data were tested for normal distribution with the Shapiro-Wilk test. The mean value of the three measurements was used for analysis. Descriptive data are presented as means and standard deviation.

### Comparison of breathing aids and body positions

Differences in terms of spatial and temporal distribution and change in EEL between spontaneous breathing, CPAP and PEP and between the different body positions within groups were assessed with paired t-tests or two way analysis of variance test with Tukey correction for multiple comparisons where applicable. Comparisons between groups (CF and healthy) were assessed with unpaired t-tests.

A p-value <0.05 was considered significant. All statistics were performed using StatsDirect, version 2.7.9 (StatsDirect Ltd., GB).

## Results

### Ventilation distribution

**Figure 1:**
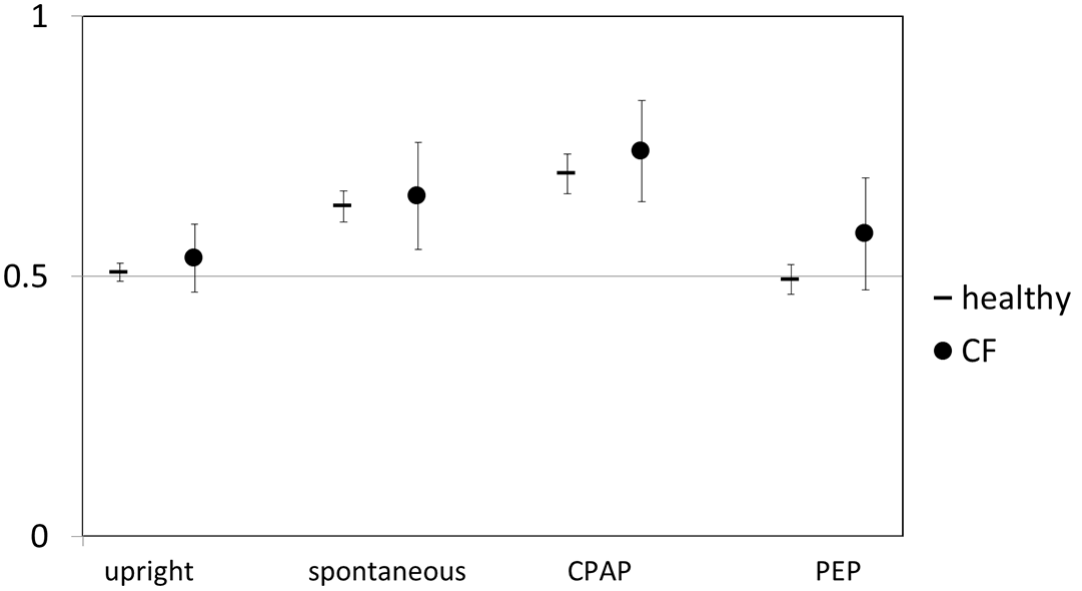
Spatial ventilation distribution with different breathing aids. Mean (95% CI) of the percentage of ventilation directed to the right lung in right lateral position for healthy and CF. For comparison spontaneous breathing in upright position is shown. Values >0.5 indicate more ventilation of the right lung, values <0.5 more ventilation of the left lung.

In upright position we found no difference in either spatial or temporal ventilation distribution between the right and left lung irrespective of the breathing aid in CF as well as in healthy. In right lateral position there was a significant shift of spatial distribution towards the right lung during spontaneous breathing in CF and healthy. This effect was enhanced by the use of CPAP and neutralised with PEP in both groups. (Figure 1) Temporal distribution showed a lag of the right lung in right lateral position (CF and healthy). This effect was reduced with CPAP and increased with PEP in healthy but no significant difference was noted in CF (Table 1).

**Table 1:** Temporal ventilation distribution: Filling index of the right lung in different body positions.

	spontaneous	CPAP	PEP
upright healthy	0.97 (0.06)	0.96 (0.04)	0.95 (0.06)
		n.s.^#^	n.s.^#^
upright CF	0.96 (0.05)	0.95 (0.06)	0.96 (0.04)
		n.s.^#^	n.s.^#^
right lateral healthy	1.08 (0.03)	1.01 (0.01)	1.21 (0.11)
	p = 0.006*	p = 0.02*	p<0.001*
	[0.11 (0.07–0.15)]^‡^	[0.05 (0.03–0.07)]^‡^	[0.26 (0.19–0.33)]^‡^
		p = 0.008^#^	p<0.001^#^
right lateral CF	1.10 (0.17)	1.05 (0.10)	1.13 (0.24)
	p = 0.021*	p = 0.035*	p = 0.042*
	[0.14 (0.09–0.19)]^‡^	[0.10 (0.03–0.17)]^‡^	[0.17 (0.02–0.32)]^‡^
		n.s.^#^	n.s.^#^

Results are given as mean (standard deviation). An index >1 indicating a lag (slower filling than the rest of the lung) and an index <1 a lead (faster filling than the rest of the lung). Mean difference (95% confidence interval) to spontaneous breathing is noted in square brackets^‡^.

p-values provided for comparison with the upright position* and for comparison with spontaneous breathing within the respective body position^#^.

n.s. not significant; CF cystic fibrosis; CPAP continuous positive airway pressure; PEP positive end-expiratory pressure.

### Additional results: End-expiratory Level (EEL) and tidal volumes

Compared to spontaneous breathing we found an increase in EEL with CPAP and a decrease in EEL with PEP. This effect was only statistically significant in the healthy group. (Table 2) Tidal volumes during PEP compared to spontaneous breathing increased significantly in upright and right lateral position in both groups. No significant differences in tidal volumes were found between spontaneous breathing and CPAP. Between CPAP and PEP differences were only significant in upright position (Table 3).

**Table 2:** Change in end-expiratory level (EEL) to spontaneous breathing in the corresponding body position expressed as percentage of the average tidal volume during spontaneous breathing in upright position.

	CPAP	PEP
upright healthy	+53 (38–68)%*	−59 (−40–−78)%*
upright CF	+66 (26–106)%	−43 (−10–−76)%
right lateral healthy	+41 (23–59)%*	−38 (−20–−56)%*
right lateral CF	+2 (−16–20)%	−0.31 (−67–5)%

Results are given as mean (95% confidence interval).

All RPT devices showed significant differences in EEL compared to spontaneous breathing in both body positions in healthy (*p<0.001) but not CF.

CF cystic fibrosis; CPAP continuous positive airway pressure; PEP positive end-expiratory pressure.

**Table 3:** Change in tidal volume expressed as percentage of the average tidal volume during spontaneous breathing in the respective body position.

	CPAP	PEP
upright healthy	11 (−1–43)%	103 (50–156)%
	n.s.*	p<0.001*
		p = 0.004^#^
upright CF	15 (−2–32)%	97 (51–143)%
	n.s.*	p<0.001*
		p = 0.007^#^
right lateral healthy	37 (23–51)%	75 (25–125)%
	n.s.*	p = 0.025
		n.s.^#^
right lateral CF	28 (−1–57)%	77 (19–135)%
	n.s.*	p = 0.029*
		n.s.^#^

Results are given as mean difference (95% confidence interval).

p-values provided for comparison with spontaneous breathing* and for comparison of CPAP and PEP^#^ in the respective body position.

## Discussion

### Summary

In healthy adults and subjects with CF gravitational effects on spatial ventilation distribution in both right and left lateral position are enhanced by CPAP but neutralised by PEP. Spatial ventilation inhomogeneity is inversely related to temporal inhomogeneity.

### Differences between breathing aids

It is well known that ventilation is increased in the dependent lung during spontaneous breathing and CPAP has been shown previously to enhance gravitational effects in healthy adults, but to our knowledge this is the first study investigating ventilation distribution during PEP therapy. [14] So far the differences between breathing aids have mainly been studied with respect to their clinical effects such as mucus clearance, improvement of gas exchange or improvement of spirometry [25]–[28].

In our study we compared two devices commonly used during RPT. It has been hypothesised that their effect on mucus clearance is caused by stabilising the airways and by enhancing gravitational effects on ventilation distribution [1], [6], [29] If it is accepted by the patient and if there are no unwanted side effects, gravity assisted positioning (without any device) as a possibility to enhance mucus clearance is recommended in patients with cystic fibrosis or non-cystic fibrosis-related bronchiectasis [30].

We demonstrated that the investigated devices had a different influence on ventilation distribution in the lateral position, with CPAP enhancing the effect of gravity on spatial distribution whereas PEP appeared to counteract it. Temporal distribution showed the opposite.

We can only speculate on the underlying mechanisms leading to the presented differences between CPAP and the PEP devices. In healthy lungs, the gravity-dependent strain on the independent parts of the lung will lead to a decrease in compliance, corresponding to a shift towards the right, flatter part of the pressure-volume curve. This decrease in compliance will tend to reduce ventilation in these parts of the lung. The application of continuous pressure during inspiration as well as expiration (CPAP) will lead to a further decrease in compliance of the independent parts of the lung and thus decreased local tidal volume. This effect has been described before. [14] As expected from the different breathing patterns used during these therapies end-expiratory level compared to spontaneous breathing was increased with CPAP and decreased with PEP, leading to different lung volumes at the beginning of inspiration. This will influence both spatial and temporal ventilation distribution as shown in healthy adults by Schnidrig et al. [11] With the combination of slightly reduced EEL (healthy −0.38; CF −0.31) and the significantly higher tidal volume (1.8 times normal in both groups) with PEP compared to spontaneous breathing we would expect a shift of ventilation to the right lung in right lateral position. This is not the case in both groups so our results cannot be explained by the change in breathing pattern during PEP therapy alone. PEP is only active during expiration, possibly leading to a homogenisation of the compliance of the lungs at the end of expiration. Similar compliance of the lungs during inspiration will minimise differences of tidal volumes directed to the dependent and independent parts of the lungs.

The much higher inter-subject variability in CF compared to healthy most likely reflects the differences in disease severity. The effect of different devices cannot be predicted by the diagnosis of CF and conventional lung function tests and EIT might help to further individualise RPT in CF.

### Strength and weaknesses of the study

To our knowledge this is the first study assessing the influence of different breathing aids on gravity-dependent ventilation distribution in healthy adults and subjects with CF. All participants were familiar with the use of the different devices. EIT itself does not influence the breathing pattern unlike other lung function techniques which use special breathing manoeuvres. With the random order of application of devices/body positions and the consistency of the measured effects we can rule out long lasting effects of one specific device, body position or a potential effect of cardiac oscillations.

Nevertheless, our study has some limitations. First, as the duration of EIT measurements was fixed at 45 seconds for all subjects, the number of breaths analysed was variable. Given the statistical analysis used (intra-subject comparison) and the highly significant differences shown in all subjects this is unlikely to be of any importance. Multiple studies have been performed to evaluate the value of EIT in assessing regional differences of ventilation in animals, infants and adults. EIT has been validated against computer tomography, ventilation scintigraphy and positron emission tomography and a variety of EIT derived indices showed a very good reproducibility. [31]–[33] In our study we compared ventilation of the right and left lung, which might have led to a certain loss of spatial information. Nevertheless, we were able to show clear differences between breathing aids.

We only investigated young adults. Thus, any conclusions drawn from this study may not apply for elderly people or very young children. This needs to be investigated in different clearly specified disease groups especially since the devices are applied on sick and usually not on healthy lungs.

Pressures for CPAP and PEP were different in CF (10 mbar) and healthy (15 mbar) which might at least partly explain the slightly lower effect of PEP in CF. Nevertheless the distinct differences between devices remain visible even with lower pressures.

Finally, in the present study we did not directly measure tidal volume, which possibly may influence ventilation distribution. We estimated tidal volume using the EIT signal normalised for tidal volume during spontaneous breathing. In right lateral position where we found the biggest differences in spatial and temporal ventilation distribution tidal volumes were only significantly different between spontaneous breathing and PEP.

### Clinical relevance

Given the importance of physiotherapy in different pulmonary diseases and the frequent use of breathing aids, it is important to understand the mechanisms of this therapy. We have demonstrated that breathing aids have different effects on ventilation distribution with a very effect size, but the therapeutic effect remains unclear. Whether PEP or CPAP is better for individual subjects needs to be evaluated in an interventional study assessing clinically relevant endpoints. Based on our results one could speculate that for CF PEP combined with lateral position might be superior for mucus clearance and CPAP combined with lateral position might be superior to overcome atelectasis. Nevertheless, EIT might help to find the optimal breathing aid for each patient and to apply individualised, patient-tailored therapy.

## Conclusion

Breathing aids show distinct differences in their effects on gravity-dependent ventilation distribution in healthy adults and CF. EIT might be used for individualised RPT in CF. In future studies individualised techniques need to be combined with measures of efficiency such as gas exchange, mucus clearance or other functional outcomes.
